# Tumorigenicity assay essential for facilitating safety studies of hiPSC-derived cardiomyocytes for clinical application

**DOI:** 10.1038/s41598-018-38325-5

**Published:** 2019-02-13

**Authors:** Emiko Ito, Shigeru Miyagawa, Maki Takeda, Ai Kawamura, Akima Harada, Hiroko Iseoka, Shin Yajima, Nagako Sougawa, Noriko Mochizuki-Oda, Satoshi Yasuda, Yoji Sato, Yoshiki Sawa

**Affiliations:** 1 Department of Cardiovascular Surgery, Osaka University Graduate School of Medicine, Suita, Osaka, 565-0871 Japan; 2 Division of Cell-Based Therapeutic Products, National Institute of Health Sciences, Kawasaki, Kanagawa 210-9501 Japan

## Abstract

Transplantation of cardiomyocytes (CMs) derived from human induced pluripotent stem cells (hiPSC-CMs) is a promising treatment for heart failure, but residual undifferentiated hiPSCs and malignant transformed cells may lead to tumor formation. Here we describe a highly sensitive tumorigenicity assay for the detection of these cells in hiPSC-CMs. The soft agar colony formation assay and cell growth analysis were unable to detect malignantly transformed cells in hiPSC-CMs. There were no karyotypic abnormalities during hiPSCs subculture and differentiation. The hiPSC markers TRA1-60 and LIN28 showed the highest sensitivity for detecting undifferentiated hiPSCs among primary cardiomyocytes. Transplantation of hiPSC-CMs with a LIN28-positive fraction > 0.33% resulted in tumor formation in nude rats, whereas no tumors were formed when the fraction was < 0.1%. These findings suggested that combination of these *in vitro* and *in vivo* tumorigenecity assays can verify the safety of hiPSC-CMs for cell transplantation therapy.

## Introduction

A large number of patients are suffering from incurable diseases in worldwide and stem cell therapy using human induced pluripotent stem cells (hiPSCs) holds promise for curing intractable diseases^1–4^. However, for the clinical application of hiPSC, it is important to identify and remove residual undifferentiated or malignant transformation cells that have potentially tumorigenic before transplantation^5–7^. Therefore, it is important to develop a highly sensitive assay for the detection of residual undifferentiated stem cells and malignant transformed cells in the transplanted cells to confirm the safety in hiPSCs therapy^8–11^.

It was recently reported that residual undifferentiated cells in hiPSCs-derived products *in vitro* can be detected by quantitative real-time polymerase chain reaction (qRT-PCR)^8^. qRT-PCR was used to detect a very small number of residual undifferentiated cells expressing LIN28 in hiPSC-derived retinal pigment epithelium (hiPSC-RPE) cells, indicating that this marker is reliable for identifying undifferentiated hiPSCs and thereby promising the safety of hiPSC therapy.

In this study, we verified whether *in vitro* tumorigenecity assay system can evaluated residual undifferentiated hiPSCs and malignant transformed cells in hiPSC-derived cardiomyocytes (hiPSC-CMs). We also verified whether this *in vitro* system can ensured the safety of hiPSC therapy by *in vivo* analysis.

## Results

### Differentiation of human iPSCs into cardiomyocyte *in vitro*

**Figure 1:**
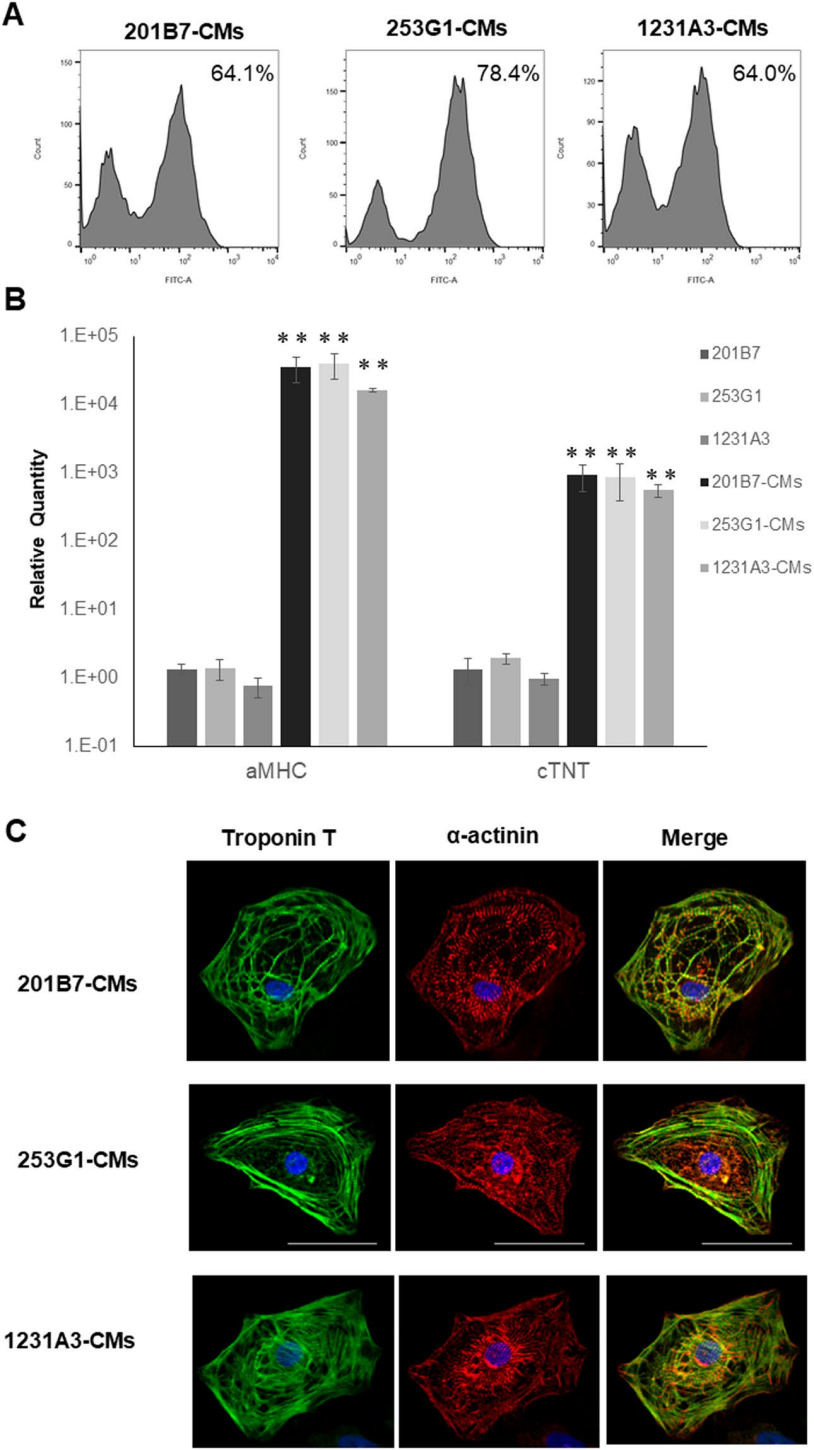
Differentiation of human iPSCs into hiPSCs-CMs *in vitro*. (**A**) Representative flow cytometry data for hiPSCs-CMs labeled with anti-cTnT antibody. (**B**) mRNA expression of *aMHC* and *cTNT* in hiPSC-CMs as compared to hiPSCs as determined by qRT-PCR. **P < 0.01. (**C**) Immunolabeling of hiPSC-CMs with anti-cTNT (green) and anti-sarcomeric α-actinin (red) antibodies with Hoechst 33342 staining. Scale bar, 50 μm.

Cardiomyocyte differentiation of three hiPSC lines (201B7, 253G1, and 1231A3 cells) was induced using a variety of factors and compounds. HiPSCs-CMs were positive for the cardiac marker cardiac troponin T (cTNT) (201B7-CMs, 64.1%; 253G1-CMs, 78.4%; and 1231A3-CMs, 64.0%), as determined by fluorescence-activated cell sorting (FACS) (Fig. 1A). A qRT-PCR analysis revealed that *cTNT* and *α-myosin heavy chain* (*aMHC*) expression increased with differentiation (Fig. 1B). Sarcomeric structures (cTNT and sarcomeric α-actinin) were observed in hiPSC-CMs by immunohistochemical analysis (Fig. 1C).

### Detection of malignantly transformed cells *in vitro*

**Figure 2:**
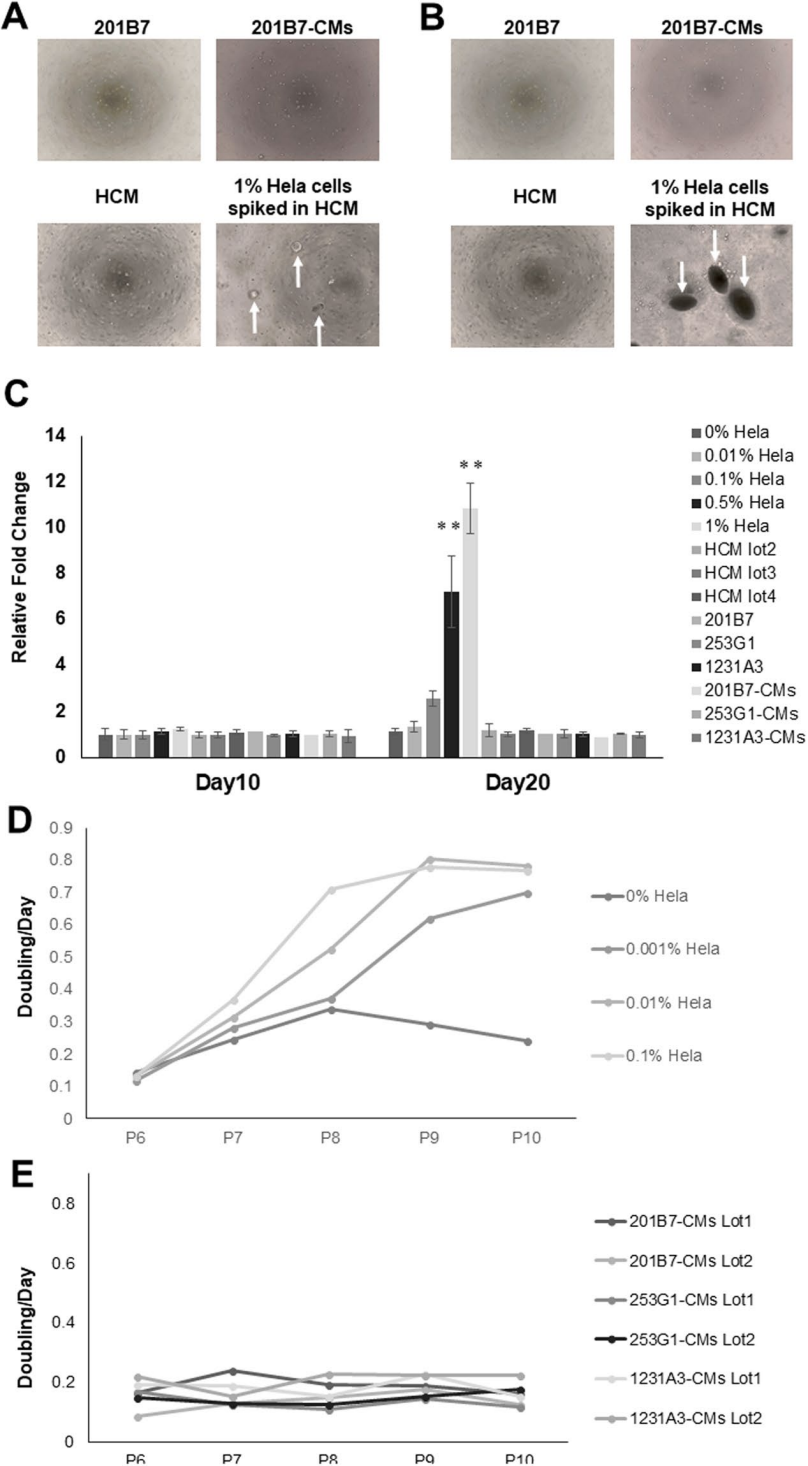
Detection of malignantly transformed cells *in vitro*. (**A**–**C**) Soft agar colony formation assay. Phase contrast micrographs of 201B7 cells, 201B7-CMs, human primary cardiomyocytes (HCM), and HeLa cells spiked into HCM (1%) cultured in soft agar medium for (**A**) 10 days and (**B**) 20 days. Arrows indicate colonies. (**C**) Quantitative analysis of fluorescence reflecting colony formation in hiPSCs, hiPSC-CMs, HCM Lot2~4, and HeLa cells spiked into HCM Lot1. Experiments were performed in triplicate. Each bar represents mean ± SD. **P < 0.01 vs. the 0% control. (**D**,**E**) Analysis of hiPSCs, hiPSCs-CMs, and HeLa cell growth and growth curves of hMSC contaminated with HeLa cells (**D**) and hiPSC-CMs (**E**).

The soft agar colony formation assay and cell growth analysis are highly sensitive methods for detecting malignantly transformed cells *in vitro*^8,10,11^. In the present study, HeLa cells used as a positive control^11–13^. Colonies were formed by HeLa cells (1%) added to 1.0 × 10^4^ human primary cardiomyocyte (HCM) after 10 days and even 0.5% HeLa cells on day 20. However, 201B7 cells, 201B7-CMs, 253G1, 253G1-CMs, 1231A3, 1231A3-CMs and HCM did not grow in soft agar (Fig. 2A–C). We also analyzed the growth rate of human mesenchymal stem cells (hMSCs 1 × 10^6^) contaminated with HeLa cells (10, 100, or 1000 cells) and hiPSC-CMs (Fig. 2D,E). The growth rate of hMSCs contaminated with HeLa cells has been reported as a control to detection of the immortalized/tumorigenic cells in human somatic cells^10^. These results indicated that the proliferation rate of HeLa cells contamination of hMSCs increased in dose dependent manner. However, the growth rate of hiPSC-CMs were constant and slow.

### Detection of undifferentiated hiPSCs *in vitro*

**Figure 3:**
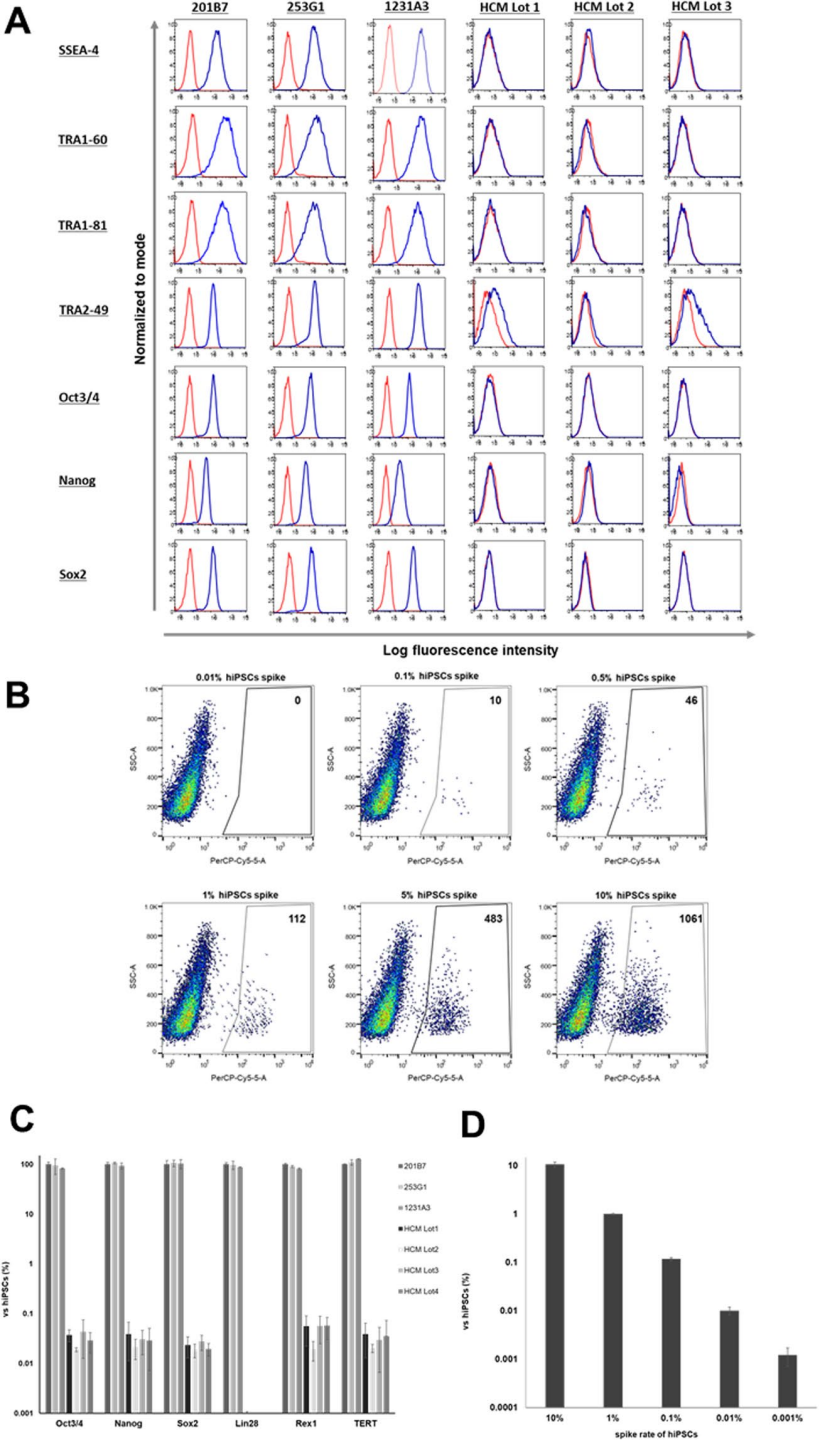
Detection of undifferentiated hiPSCs *in vitro*. (**A**,**B**) Flow cytometry analysis of hiPSCs and primary cardiomyocytes *in vitro*. (**A**) Flow cytometry data of hiPSCs and primary cardiomyocytes labeled with antibodies against a variety of stem cell markers including Oct3/4, Nanog, Sox2, SSEA-4, TRA1-60, TRA1-81, and TRA2-49. (**B**) 201B7 cells were spiked into primary cardiomyocytes at concentrations of 10%, 5%, 1%, 0.5%, 0.1%, and 0.01% and analyzed by flow cytometry with anti-TRA 1-60 antibody. (**C**) mRNA expression levels of *Oct3/4*, *Nanog*, *Sox2*, *Rex1*, and *telomerase reverse transcriptase* (*TERT*) in hiPSCs and primary cardiomyocytes, as detected by qRT-PCR. (**D**) 201B7 cells were spiked into primary cardiomyocytes at concentrations of 10%, 1%, 0.1%, 0.01%, and 0.001% and *LIN28* mRNA level was evaluated by qRT-PCR.

It was recently reported that undifferentiated hiPSCs spiked in primary RPE cells can be detected by FACS and qRT-PCR^8^. To identify the most sensitive markers for detection of residual undifferentiated cells in a population of hiPSC-CMs, primary cardiomyocyte spiked with hiPSCs were analyzed by FACS and qRT-PCR. We first examined the expression of stem cell markers such as octamer-binding protein (Oct)3/4, Nanog, (sex-determining region)-box (Sox)2, stage-specific embryonic antigen (SSEA)-4, TRA1-60, TRA1-81, and TRA2-49 by FACS. All of these markers were expressed by undifferentiated hiPSCs. Oct3/4, Sox2, SSEA-4, TRA1-60, and TRA1-81 expression distinguished hiPSCs from primary CMs (Fig. 3A). It has been reported that TRA 1-60 is a marker for embryonal carcinoma and it has also been used to detect undifferentiated hiPSCs among primary RPE cells^8^. We therefore used anti-TRA-1-60 antibody to detect undifferentiated hiPSCs in populations of primary CMs spiked with 10%, 5%, 1%, 0.5%, 0.1%, and 0.01% 201B7 cells by FACS, and found that the detection limit was 0.1% (Fig. 3B). We also compared mRNA levels of *Oct3/4*, *Nanog*, *Sox2*, *LIN28*, and *Rex1* in hiPSCs and primary cardiomyocyte by qRT-PCR to identify selective markers for undifferentiated hiPSCs. *LIN28* was expressed in hiPSCs but not in primary cardiomyocyte (Fig. 3C). The limit of detection of *LIM28* mRNA in primary cardiomyocyte spiked with 1%, 0.1%, 0.01%, and 0.001% 201B7 cells was 0.001% by qRT-PCR (Fig. 3D).

### Karyotype analysis

**Figure 4:**
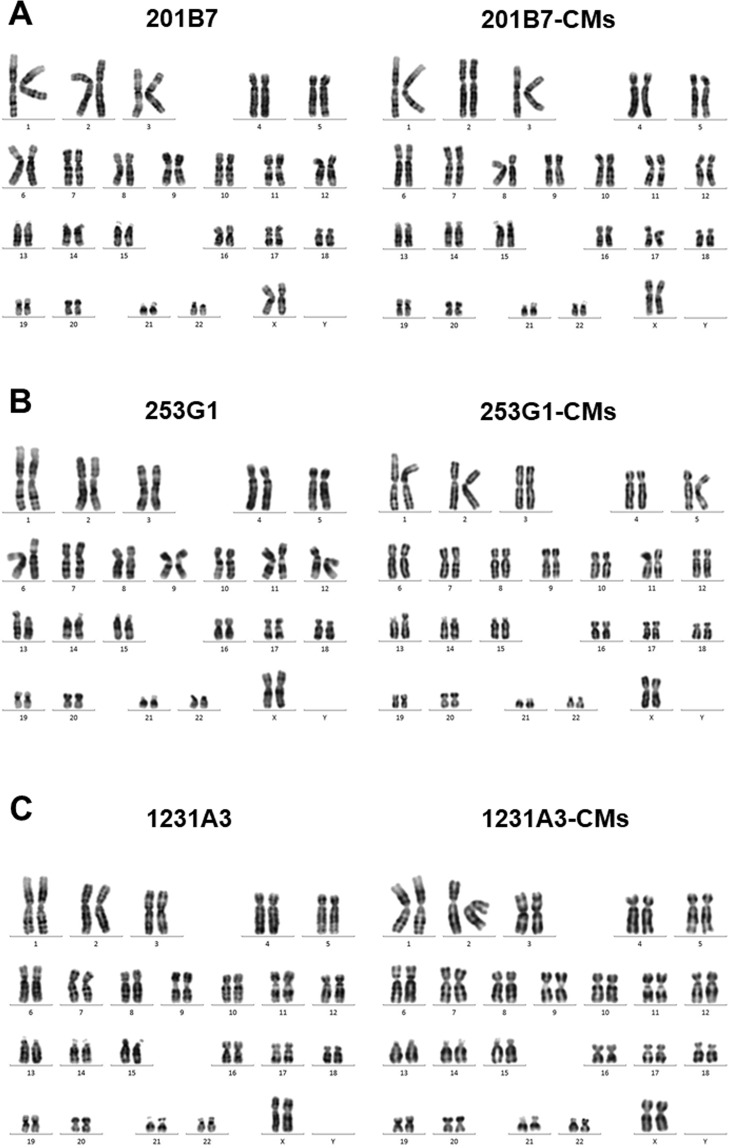
Karyotype analysis. Representative karyograms of (**A**) 201B7 cells and 201B7-CMs, (**B**) 253G1 cells and 201B7-CMs, (**C**) 1231A3 cells and 1231A3-CMs.

We carried out a karyotype analysis in order to assess genetic alterations during hiPSC subculture and differentiation. It has been reported that the risk of aberrant hiPSC karyotypes increases with passage number; we therefore examined late-passage hiPSCs and hiPSC-CMs. There was no karyotypic aberrations in CMs derived from 20B7, 253G1 and 1231A3 cells during hiPSC subculture and differentiation (Fig. 4).

### Detection of undifferentiated hiPSCs *in vivo*

**Figure 5:**
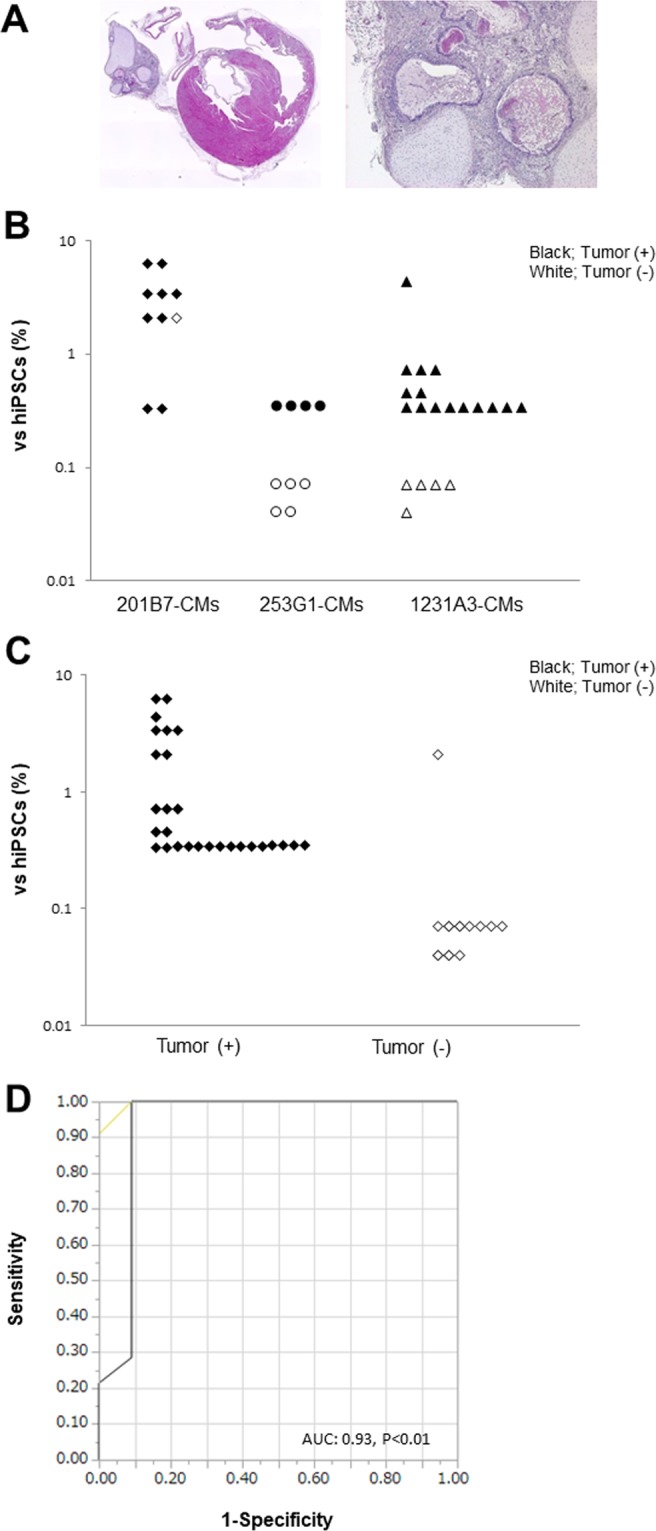
Detection of undifferentiated hiPSCs *in vivo*. Transplantation of hiPSC-CM sheets into the left ventricular surface of immunodeficient rats. (**A**) Representative H&E staining of teratoma. (**B**) Relationship between *LIN28* mRNA expression in hiPSC-CMs by cell line and tumor formation. (**C**) Relationship between *LIN28* mRNA expression in hiPSC-CMs and tumor formation. (**D**) ROC curves for *LIN28* mRNA expression in all hiPSC-CMs and tumor formation.

Immunodeficient rodents such as nude mice (BALB/cAJcl-nu/nu), SCID mice (C.B-17/Icr-scid/scidJcl), NOD-SCID mice (NOD/ShiJic-scidJcl), NOG mice (NOD/Shi-scid,IL-2RγKO Jic), and nude rats (F344/NJcl-rnu/rnu) have been widely used for tumorigenicity tests^14,15^. We transplanted cell sheets derived from hiPSC-CMs into the heart of nude rats in order to assess their tumorigenic potential. After 4 months, tumor-bearing nude rats were sacrificed, and the tumor were assessed by hematoxylin and eosin (HE) staining. The tumors observed were teratomas (Fig. 5A). Transplantation of hiPSC-CMs containing a LIN28-positive fraction >0.33% induced tumor formation in 28/29 nude rats, whereas a LIN28-positive fraction <0.1% failed to induce tumor formation (0/10 rats) (Fig. 5B,C). A receiver-operating characteristic (ROC) curve analysis revealed that there was no tumor formation by hiPSC-CMs with a LIN28-positive fraction <0.33% (area under the ROC curve = 0.93, P < 0.01) (Fig. 5D).

## Discussion

Although hiPSC-CMs can potentially be used to treat severe heart failure, tumorigenicity limits their clinical application. Detecting and removing residual iPSCs or differentiated CMs that have undergone malignant transformation may be a key target to promise can ensure the safety of iPSC therapy. In this study, we established an assay for detection the potential tumorigenic cells in hiPSC-CMs *in vitro* and *in vivo*.

Although cervical cancer (HeLa) cells formed colonies in the soft agar colony formation assay, hiPSC-CMs did not exhibit colony formation. Moreover, cell growth analysis revealed that, HeLa cells proliferated notably faster compared with hiPSC-CMs having slow growth, therefore malignantly transformed cells were not detected in the *in vitro* assay of hiPSCs. TRA 1-60 and LIN28 are ideal markers for distinguishing residual undifferentiated hiPSCs among hiPSC-CMs by FACS and qRT-PCR. The latter was the more sensitive detection method of residual undifferentiated hiPSCs in hiPSCs-CMs. In the spike test, the detection limit was 0.001% by qRT-PCR as compared to 0.1% by FACS. In karyotype test, No karyotypic abnormalities were observed during hiPSC culture and cardiomyocyte differentiation. Additionally, *in vivo* tumorigenicity test, the mRNA expression of *LIN28*—a marker of undifferentiated hiPSCs—was associated with tumorigenesis in hiPSC-CMs transplanted into the heart of T cell-deficient nude rats.

There are a few possible mechanisms by which tumorigenesis can occur in iPSC-based therapy. In addition to teratomas, tumors may form from cells with tumorigenicity that have undergone malignant transformation during hiPSC culture and cardiomyocyte differentiation. The focus formation test, soft agar colony formation assay, and cell growth assay are widely used methods for detecting malignant transformed cells^8,10,11^. In the present study, we did not detect any malignantly transformed cells in the hiPSC-CMs using combination of soft agar colony formation assay and cell growth assay, providing one of evidences for the safety these cells.

Tumors can also arise from residual undifferentiated cells^14,16–18^. A correlation between residual undifferentiated cells and teratoma formation after transplantation of neurospheres derived from mouse iPSCs has been reported^6^. It is necessary to use optimal stem cell markers for detecting residual undifferentiated cells depending on the differentiated cell type. Markers expressed also in target cells after differentiation cannot accurately detect residual undifferentiated cells in hiPSCs-derived products. Kuroda *et al*. have reported a method to detect residual undifferentiated cells in hiPSCs-derived retinal pigment epithelium cells (hiPSC-RPE cells) using Lin 28 in qRT-PCR^8^. LIN28 is involved in maintaining the self-renewal ability of stem cells, and it is one of the initialization factors used for inducing iPS cells from human somatic cells. As such, Lin 28 is highly expressed in human iPSCs. The results of our study strongly suggest that LIN28 is a marker that can be useful for detecting undifferentiated residual cells in hiPSC-CMs by qRT-PCR, as it showed the highest sensitivity of all the Stem cell markers tested, with no expression in terminally differentiated cardiomyocytes and highly expression in hiPSCs.

In addition to residual undifferentiated cells or malignant transformed cells, critical genomic changes and the survival of foreign genes in iPS cells have some possibilities for tumor formation in hiPSCs-derived products. The karyotype analysis, which has capability in detection of extremely large genetic abnormalities, revealed no major genomic changes in hiPSC-CMs. To check genetic abnormality in detail a SNP array *et al*., which can analyze small or large genomic variations in whole genome sequence with extremely high sensitivity, may be critical for confirming the safety of hiPSCs-derived products for therapeutic applications. However, the correlation between genomic abnormalities and tumorigenicity remains to be determined. A more detailed analysis of these would require further study.

The results of our study demonstrate that hiPSCs-derived products with high LIN28 mRNA expression exhibited a higher rate of tumorigenesis, indicating that LIN 28 can predict tumorigenesis after transplantation of hiPSC-CM. However, this must be confirmed in different type of immunodeficiency animal model, routes of administration, methods of transplantation (e.g., with Matrigel), gender and number of animals, dose of cells, monitoring periods etc.

Our system for screening tumorigenic iPSC derivatives composed of *in vitro* and *in vivo* assays which asses tumorigenicity of malignant transformed cells and LIN28-positive cells, respectively. However, *in vivo* tumorigenicity assays are costly and time-consuming. Moreover, some degree of skill is required to transplant cells into rat or mouse heart. We suggest that *in vitro* assays which detect the malignant transformed cells and LIN28 expression level may be substituted for *in vivo* assays.

In conclusion, we developed an assay that combines *in vitro* quantification of tumorigenic cells and *in vivo* tumorigenicity assessment to verify the safety of hiPSC-derived CMs for regenerative therapy of heart failure or heart disease. Further studies are warranted to verified whether this *in vitro* system can ensured the safety of hiPSC therapy for the clinical application of cell transplantation therapy using human iPSC-CMs.

## Experimental Procedures

Animal experiments were performed according to the Guide for the “Care and Use of Laboratory Animals” (National Institutes of Health publication). Experimental protocols were approved by the Ethics Review Committee for Animal Experimentation of Osaka University Graduate School of Medicine (reference number; 25-025-034).

### Human iPSC cultures

The 201B7 cells (four factors: Oct3/4, Sox2, Kruppel-like factor [Klf]4, and c-Myc) and 253G1 cells (three factors: Oct3/4, Sox2, and Klf4) hiPSC lines were purchased from RIKEN Bioresource Center (Ibaraki, Japan). The hiPSC cell line 1231A3 cells (six factors: Oct3/4, Sox2, Klf4, L-Myc, LIN28, and p53-short hairpin RNA) generated from human mononuclear cells was a gift from Professor Yamanaka (Kyoto University, Kyoto, Japan). The 201B7 cells were maintained as previously described^19^; 253G1 cells were maintained according to Matsuura *et al*.^20^; and 1231A3 cells were cultured on iMatrix511 (Nippi, Tokyo, Japan)-coated dishes in Stem Fit Ak03N (Ajinomoto, Tokyo, Japan).

### Cardiomyocyte differentiation and purification

#### Cardiomyocyte differentiation and purification. 

Cardiomyogenic differentiation of 201B7 and 253G1 cells was induced as previously described^19,20^. To differentiate 1231A3 cells into the cardiac lineage, we used a modified version of an established protocol^21^. Briefly, embryoid bodies (EBs) were generated in an EZ sphere (Iwaki, Shizuoka, Japan) in Stem Fit Ak03N without basic fibroblast growth factor (bFGF; R&D Systems, Minneapolis, MN, USA) in the presence of 10 μM Y-27632 (Rho-associated protein kinase inhibitor; Wako Pure Chemical Industries, Osaka, Japan) and bone morphogenetic protein (BMP)-4 (R&D Systems). After EB formation, the culture medium was replaced with differentiation medium containing Stem Fit Ak03N without bFGF and supplemented with several human recombinant proteins including BMP-4 and activin A, bFGF, and vascular endothelial growth factor (all from R&D Systems) as well as the small molecule inhibitor of Wnt production 3 (Stemgent, Lexington, MA, USA), SB431542 (Sigma-Aldrich, St. Louis, MO, USA) and Dorsomorphin (Sigma-Aldrich). After cardiac differentiation, the cells were dissociated with 0.25% trypsin/EDTA (Thermo Fisher Scientific, Waltham, MA, USA), cells were plated onto the gelatin coated dish. HiPSC-CMs were maintained in Dulbecco’s modified Eagle’s medium (DMEM; Nacalai Tesque, Kyoto, Japan) containing 10% fetal bovine serum (FBS; Sigma-Aldrich) or DMEM without methionine (Thermo Fisher Scientific) for eliminate residual undifferentiated cells^22^.”

### Flow cytometry

For intracellular staining, cells were fixed with CytoFix fixation buffer (BD Biosciences, Franklin Lakes, NJ, USA), permeabilized with Perm/Wash buffer (BD Biosciences), then labeled with directly conjugated peridinin chlorophyll (PerCP)-Cy5.5 mouse anti-OCT4(BD Biosciences), phycoerythrin (PE) mouse anti-NANOG (BD Biosciences), Alexa Fluor 647 anti-SOX2 (BD Biosciences), and unconjugated mouse anti-cardiac isoform of Troponin T (cTnT) (Santa Cruz Biotechnology, Dallas, TX, USA) in Perm/Wash buffer. Alexa Fluor 488 goat anti-mouse IgG (Thermo Fisher Scientific, Waltham, MA, USA) was used as a secondary antibody. For surface labeling, cells were incubated with directly conjugated PE anti-SSEA-4 (BD Biosciences), PerCP-Cy5.5 anti-TRA-1-60, PerCP-Cy5.5 anti-TRA-1-81, and Alexa Fluor 488 anti-TRA-2-49 (BioLegend, San Diego, CA, USA). The cells were analyzed by FACS Canto II system (BD Biosciences), and data were analyzed with FlowJo software (Tree Star, Ashland, OR, USA).

### RNA extraction and qRT-PCR

Total RNA was extracted from hiPSCs and hiPSC-CMs using the PureLink RNA Mini kit (Thermo Fisher Scientific) and used to synthesize cDNA with the Super Script III First-strand Synthesis System and random primers (Thermo Fisher Scientific). qRT-PCR was performed on a ViiA 7 RT-PCR system (Thermo Fisher Scientific) using SYBR Green and human stem cellspecific primers^8^ (Table 1). Each sample was analyzed in triplicate for each gene studied. The average copy number of gene transcripts was measured relative to the level of glyceraldehyde 3-phosphate dehydrogenase in each sample.

**Table 1:** Forward and reverse primers for real time PCR experiments.

Gene	Direction	Sequence
aMHC	Forward	5′-GGGATAACCAGGGGAAGCACCAAGA-3′
	Reverse	5′-TGCCTCCCTCCCGGGACAAAAT-3′
cTNT	Forward	5′-AAAGCCCAGGTCGTTCATGCCC-3′
	Reverse	5′-CATTCCGGATGCGCTGCTGC-3′
Oct3/4	Forward	5′- GAAACCCACACTGCAGCAGA -3′
	Reverse	5′- TCGCTTGCCCTTCTGGCG -3′
Nanog	Forward	5′- CTCAGCTACAAACAGGTGAAGAC -3′
	Reverse	5′- TCCCTGGTGGTAGGAAGAGTAAA -3′
Sox2	Forward	5′- GCGCCCTGCAGTACAACTC -3′
	Reverse	5′- CGGACTTGACCACCGAACC-3′
Lin28	Forward	5′- CACGGTGCGGGCATCTG -3′
	Reverse	5′- CCTTCCATGTGCAGCTTACTC -3′
Rex1	Forward	5′- CCATCGCTGAGCTGAAACAAA -3′
	Reverse	5′- CCTCCAGGCAGTAGTGATCTG -3′
TERT	Forward	5′- CCTGTTTCTGGATTTGCAGGTG -3′
	Reverse	5′- GCACACATGCGTGAAACCTG -3′
GAPDH	Forward	5′-CAATGACCCCTTCATTGACC-3′
	Reverse	5′-TTGATTTTGGAGGGATCTCG-3′

### Immunofluorescence analysis

HiPSC-CMs were fixed with 4% paraformaldehyde and labeled with antibodies against cTNT (Abcam, Cambridge, UK) and anti-sarcomeric alpha actinin (α-actinin (Sigma-Aldrich) as primary antibodies, followed by secondary antibodies such as Alexa Fluor 488- or AlexaFluor555 conjugated donkey anti-rabbit or -mouse IgG (Thermo Fisher Scientific)antibodies. Nuclei were counterstained with Hoechst 33342 (Dojindo, Kumamoto, Japan). Images of samples were acquired on a confocal microscope (Fluoview FV10i; Olympus, Tokyo, Japan).

### Soft agar colony formation assay

The soft agar colony formation assay was performed using the CytoSelect 96-well cell transformation assay kit (Cell Biolabs, San Diego, CA, USA). Human cervical carcinoma cell line HeLa cells (JCRB Cell Bank, NIBIOHN, Osaka, Japan) were used as a positive control since they are known to form colonies in soft agar medium (Kusakawa *et al*.^11^; Ke *et al*.^12^; Seo *et al*.^13^). The cells were maintained in Eagle’s minimum essential medium (Sigma-Aldrich) supplemented with 10% FBS (Sigma-Aldrich) and 0.1 mM non-essential amino acids (Thermo Fisher Scientific). The primary human cardiomyocytes (HCM) (PromoCell, Heidelberg, Germany) were were maintained in Myocyte Growth Medium (PromoCell). HeLa cells (1%, 100 cells; 0.5%, 50 cells; 0.1%, 10 cells; and 0.01%, 1 cell) were spiked into 1.0 × 10^4^ HCM and grown in soft agar for 10 or 20 days. The cells were lysed and absorbance was recorded on a microplate reader (DS Pharma Biomedical, Osaka, Japan) at a wavelength of 485/520 nm filter set. The experiment was performed in triplicate.

### Cell growth assay

Cell growth assay of hiPSCs was evaluateld according to Kono *et al*.^10^. Human mesenchymal stem cells (hMSCs) (Lonza, Walkersville, MD, USA) were maintained in mesenchymal stem cell growth medium bullet kit (Lonza). A total of 1 × 10^6^ hMSCs were mixed with 1000, 100, or 10 of HeLa cells and used as a positive control. HiPSCs-CMs were dissociated with TrypLE Select (Thermo Fisher Scientific) and 1 × 10^6^ hiPSC-CMs were seeded in T175 flasks (Corning Inc., Corning, NY, USA) and cultured. This process was repeated for 10 passages and growth rate was calculated.

### Karyotype analysis

Thymidine was added to hPSCs-CMs cultured at 37 °C under 5% CO_2_ and cultures were synchronized from 10 to 24 h. After washing to remove the thymidine, colcemid was added and culture synchronization was carried out for 6 to 24 h to obtain metaphase cells, which were fixed and added as a drop onto a slide glass to prepare chromosome specimens. The preparation was stained with Hoechst 33258 and quinacrine mustard solution to obtain Q bands. Chromosomes were classified according to band pattern and karyograms were prepared. A karyotype of up to 20 cells was analyzed in the chromosome karyogram.

### HiPSC-CM sheet transplantation into nude rats

Male F344/NJclrnu/rnu rats (5–6 weeks old, weighing 190–250 g) were obtained from CLEA Japan (Tokyo, Japan). A total of 1 × 10^7^ cells per hiPSC-CMs sheet was transplanted into the left ventricular surface of each rat heart. Rats were sacrificed 4 months later. All surgeries and sacrifices were performed on deeply anesthetized animals.

### Histological analysis

Heart specimens of rats with transplanted hiPSC-CMs were fixed with 10% buffered formalin and embedded in paraffin. Serial paraffin-embedded sections cut at a thickness of 2 μm were deparaffinized in xylene, dehydrated in a graded series of ethanol, and stained with hematoxylin and eosin. The sections were assessed under a light microscope (Leica, Wetzlar, Germany).

### Statistical analysis

All results of *in vitro* assays are presented as mean ± standard deviation (SD) and were analyzed with the Student’s t test. P values < 0.05 were considered statistically significant. Receiver-operating-characteristic (ROC) analysis was performed using JMP v.12 software (SAS Institute, Cary, NC, USA).
